# Delayed cerebral metastasis after complete resection of left atrial cardiac myxoma: a case report

**DOI:** 10.1093/omcr/omag081

**Published:** 2026-05-24

**Authors:** Shogo Narikiyo, Toru Yasutsune, Masayoshi Umesue

**Affiliations:** Department of Cardiovascular Surgery, Matsuyama Red Cross Hospital, 1 Bunkyo-cho, Matsuyama, Ehime 790-8524, Japan; Department of Cardiovascular Surgery, Matsuyama Red Cross Hospital, 1 Bunkyo-cho, Matsuyama, Ehime 790-8524, Japan; Department of Cardiovascular Surgery, Matsuyama Red Cross Hospital, 1 Bunkyo-cho, Matsuyama, Ehime 790-8524, Japan

**Keywords:** cardiac myxoma, cerebral metastasis, embolic infarction

## Abstract

Cardiac myxoma (CM) is the most common benign primary cardiac tumor and is usually curable with complete surgical excision. However, CM can cause systemic embolization, and delayed cerebral metastasis is extremely rare. A 54-year-old woman presented with progressive lower limb weakness and was diagnosed with multiple cerebral infarctions associated with a left atrial pedunculated tumor. The tumor was completely resected under cardiopulmonary bypass and confirmed histopathologically as CM. Seven months later, she developed transient visual disturbances and right-hand weakness. Brain magnetic resonance imaging revealed enlarging lesions inconsistent with vascular territories. Craniotomy and histopathology with calretinin positivity confirmed metastatic myxoma. She underwent stereotactic radiotherapy and remained recurrence-free at 1 year. Even after complete resection of CM, delayed cerebral metastasis may occur. Persistent neurological surveillance and multidisciplinary management are essential for early detection and favorable long-term outcomes in patients with CM.

## Introduction

Cardiac myxoma (CM), the most common benign primary cardiac tumor, typically originates in the left atrium (LA) [1, 2]. Although histologically benign, CM can cause intracardiac obstruction, constitutional symptoms such as fever, and systemic embolization. Surgical resection is generally curative; however, CM can also lead to rare delayed central nervous system (CNS) manifestations, including cerebral metastasis and myxomatous aneurysms [3–5]. Epidemiological data suggest that while CM is benign, intracranial implantation occurs in less than 2% of cases [5]. Differentiating cerebral embolic infarction from metastatic brain lesions is crucial because their medical management differs considerably. We report a case of a patient in whom intracranial lesions progressed after LA CM resection, emphasizing that late progressive or hemorrhagic intracranial lesions should raise suspicion for tumor implantation rather than being attributed solely to embolic infarction.

## Case report

A 54-year-old woman experienced intermittent bilateral lower-limb weakness that progressed over 2 months, resulting in gait disturbance. Two days before referral, she was hospitalized, but computed tomography (CT) of her head and spine revealed no abnormalities. Symptoms partially improved, and she was discharged.

**Figure 1:**
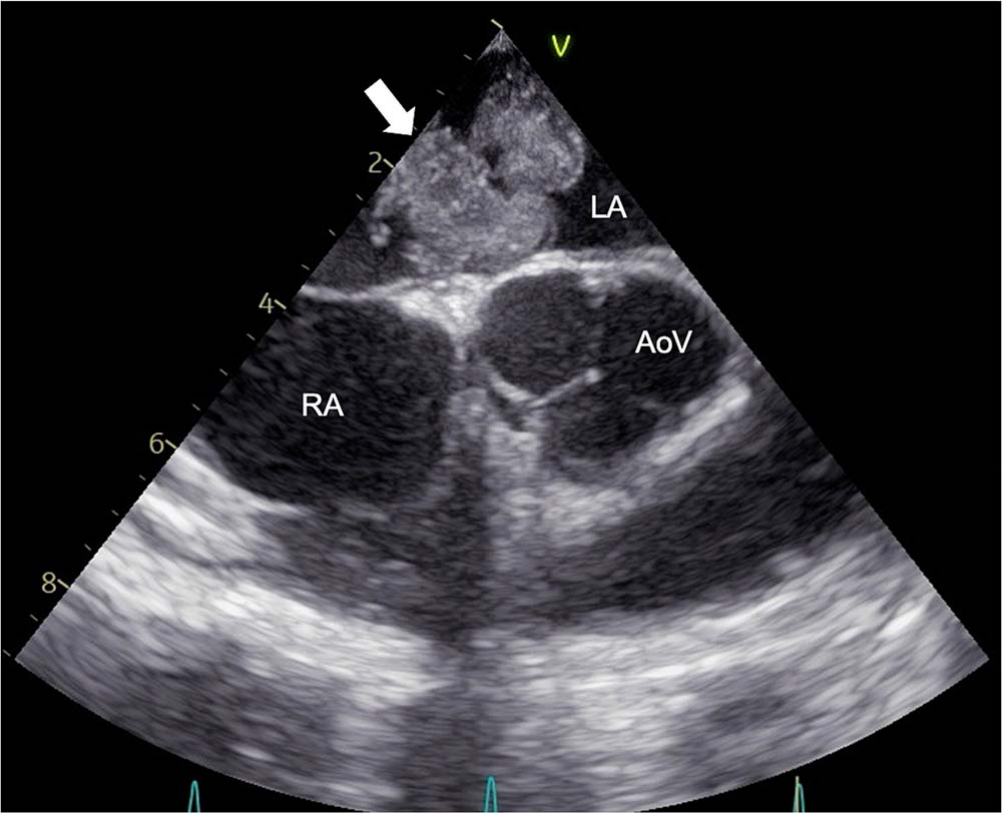
Preoperative echocardiography. Transesophageal echocardiography showing a 40-mm pedunculated tumor (arrowed) attached to the LA side of the atrial septum. RA, right atrium; LA, left atrium; AoV, aortic valve.

**Figure 2:**
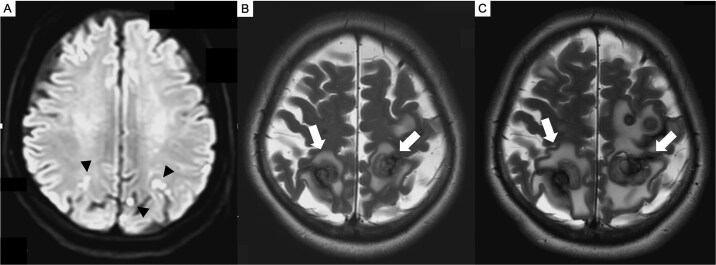
Brain MRI. (A) Preoperative diffusion-weighted imaging (DWI) showing multiple acute cerebral infarcts (arrowed) in the parietal lobe. (B) MRI (T2-weighted imaging) at 7 months postoperatively showing multiple new enhancing intracranial lesions (arrowed) corresponding to previous infarcts sites in the parietal lobe. (C) MRI (T2-weighted imaging) at 8 months postoperatively showing progressive enlargement and edema (arrowed).

The following day, she developed fever and nausea. On evaluation, she had a temperature of 38.9°C and an elevated C-reactive protein level (24 mg/dL), but neurological examination was unremarkable. Non-contrast CT demonstrated acute cerebral infarcts, a hypodense LA mass, and possible left renal and splenic infarctions. Transthoracic echocardiography revealed a mobile heterogeneous LA mass, and transesophageal echocardiography confirmed a 40-mm pedunculated mass attached to the interatrial septum (Fig. 1). Brain magnetic resonance imaging (MRI) revealed multiple acute infarcts and microbleeds (Fig. 2A).

Although modified Duke criteria were not fully met and no clear vegetations were identified on echocardiography, empiric antibiotics were initiated for suspected infective endocarditis. Blood cultures on hospital days 1 and 4 remained negative, and a follow-up CT on day 7 showed no enlargement of the known hemorrhage.

**Figure 3:**
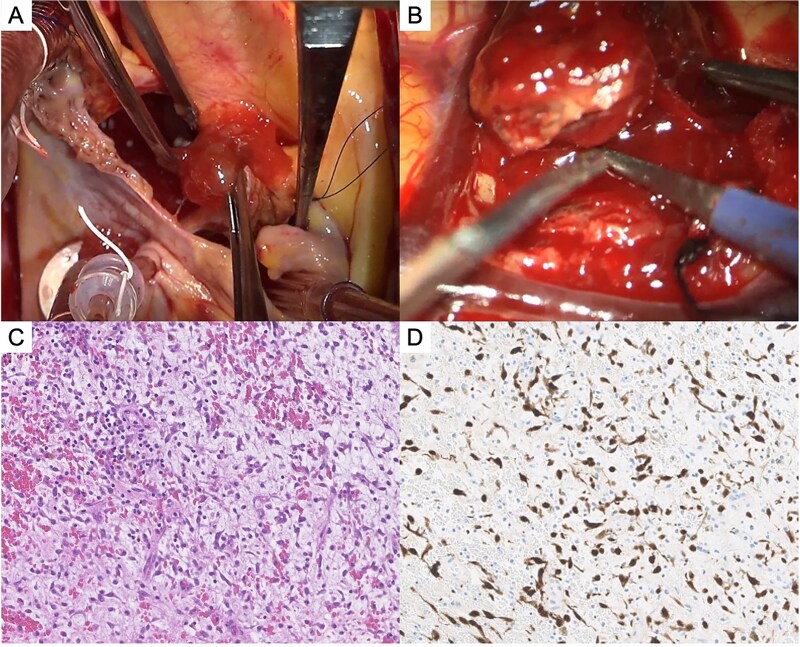
Tumor findings. (A) Intraoperative photograph of left atrial myxoma. (B) Intraoperative photograph of resected frontal lobe metastatic lesion. (C) Histopathology of cerebral metastatic myxoma (H&E, ×20). (D) Histopathology of cerebral metastatic myxoma (calretinin immunostaining, ×10).

On hospital day 12, surgical resection was performed via a trans-septal approach. A 40-mm fragile, gelatinous, pedunculated mass attached to the roof of the LA was completely resected along with the adjacent endocardial tissue (Fig. 3A). Histopathological examination revealed stellate cells within the myxoid stroma, confirming the presence of CM without malignancy, surface thrombus, or necrosis. The postoperative course was uneventful, and echocardiography confirmed complete resection with no residual atrial shunt. She was discharged on postoperative day 15.

Six months postoperatively, follow-up transthoracic echocardiography revealed no recurrence of the cardiac tumor. Seven months after the surgery, she developed transient left homonymous hemianopia. Brain MRI revealed multiple new enhancing space-occupying lesions corresponding to the preoperative infarct locations (Fig. 2B). Interleukin-6 (IL-6) levels were within the normal range. One month later, right-hand fine motor impairment developed, and MRI showed enlargement of multiple lesions with surrounding edema (Fig. 2C). The frontal lobe lesion was removed via craniotomy to obtain a definitive diagnosis and relieve mass effect. Intraoperative findings revealed an elastic tumor with intertumoral hemorrhage (Fig. 3B). Histopathology confirmed metastatic CM (Fig. 3C), with positive calretinin staining (Fig. 3D). She subsequently underwent intensity-modulated stereotactic radiotherapy (total 36 Gy in 12 fractions). Recurrent seizures and motor disturbances were controlled with antiepileptic therapy, and rehabilitation continued. At 1-year follow-up, no cardiac recurrence or new CNS lesions were observed.

## Discussion

CM accounts for approximately half of primary cardiac tumors [1]. A complete resection generally yields a recurrence rate below 5% [2]. Systemic embolization occurs in 30%–40% of cases due to tumor fragmentation or surface thrombus formation [3]. Although rare, brain metastasis is clinically significant [4, 5]. Recognizing the possibility of brain metastasis is essential for clinicians because delayed diagnosis can lead to irreversible neurological decline.

Risk factors for embolization include an irregular tumor surface, atypical tumor location (not the interatrial septum in the LA), and a narrow tumor base [6]. Rapid tumor growth or infection may further increase the embolic risk [7–9]. Interleukin-6 is a key cytokine produced by CM, often correlating with inflammatory activity and embolic potential [1]. In this case, the tumor surface was irregular, soft, and attached to the roof of the LA. The gelatinous pedunculated tumor likely contributed to the initial cerebral infarction. However, the subsequently enlarging lesions suggested metastatic spread rather than simple embolism. Patients with friable tumors should thus be monitored for not only early embolic complications but also delayed metastatic behavior, which may mimic primary brain tumors or ischemic sequelae.

Differentiating brain infarction from metastasis relies on both clinical course and imaging features. On brain MRI, brain infarcts typically conform to vascular territories and evolve towards atrophy, whereas intracranial metastatic lesions demonstrate progressive enlargement, ring enhancement, and disproportionate vasogenic edema. In this case, MRI showed an infarction before surgery and metastases afterward. Most of the metastatic lesions corresponded to the preoperative infarct locations on MRI.

Histopathological findings of myxomas show abundant myxoid stroma containing scattered spindle-shaped and stellate tumor cells, with the diagnosis confirmed by calretinin immunostaining [7, 10]. Viable myxoma cells may enter the systemic circulation through tumor fragmentation or intravascular invasion. Embolic injury to the blood–brain barrier may create a localized environment facilitating the implantation and proliferation of viable myxoma cells (‘vascular niche’ hypothesis) [4, 5, 7]. In this case, both the cardiac and intracranial tumors showed histopathological findings consistent with myxoma, leading to a diagnosis of metastatic CM.

Symptomatic solitary or limited lesions are often surgically resected, followed by stereotactic or fractionated radiotherapy. Multiple or deep-seated lesions may be treated primarily with radiotherapy; chemotherapy is reserved for selected progressive or recurrent cases [4, 5, 7, 10]. In this case, although multiple lesions were present, the frontal lobe lesion was resected to obtain a definitive metastasis diagnosis. Radiotherapy was administered to the remaining lesions to relieve symptoms. Management should be individualized based on lesion number, location, and neurological symptoms.

Long-term follow-up should include echocardiography to monitor cardiac tumor recurrence, and neurological evaluation and brain imaging should be performed where indicated. High suspicion should be maintained when new neurological deficits occur after CM surgery, as these may indicate brain metastasis rather than recurrent infarction, underscoring the importance of continued neurological vigilance despite complete tumor resection.
